# Data of the multi-wave population-based prospective Victims in Modern Society (VICTIMS) study on potential traumatic events, social support, mental health, coping self-efficacy and services use

**DOI:** 10.1016/j.dib.2024.110346

**Published:** 2024-03-19

**Authors:** Peter G. van der Velden, Carlo Contino, Marcel Das

**Affiliations:** a Tranzo, Tilburg School of Social and Behavioral Sciences, Tilburg University, PO Box 90153, Tilburg 5000 LE, The Netherlands; b Centerdata, PO Box 90153, Tilburg 5000 LE, The Netherlands; c Fonds Slachtofferhulp, PO Box 93166, Den Haag 2509 AD, The Netherlands; d Tilburg School of Economics and Management, Tilburg University, PO Box 90153, Tilburg 5000 LE, The Netherlands

**Keywords:** Trauma, Crime, PTSD, Anxiety and depression, Emotional support, Esteem support, Social acknowledgement, LISS panel

## Abstract

We present the data of the first six annual surveys of the large prospective population-based Dutch VICTIMS-study that started in 2018. Each survey systematically examines exposure to potential traumatic events in the past 12 months, including time of event and amount of stress during the event. Furthermore, each survey assesses anxiety and depression symptomatology, lack of social support, physical, mental, work, partner/family, religious, legal, administrative and financial problems, and problem-related services use. Among the by potentially traumatic events (PTEs) affected respondents, current PTSD-symptomatology, social acknowledgement, events-related contacts with various professional, and coping self-efficacy related to the (most stressful) PTE in the past 12 months was examined.

This multi-wave study is conducted using the Dutch Longitudinal Internet studies for the Social Sciences panel (LISS panel) that is based on a large traditional probability sample of the Dutch population (16+). People cannot register themselves as a panel member which minimizes the risks of selection bias. Panel members receive a financial compensation for each completed questionnaire, which has a positive effect on the response rate. Households that would otherwise be unable to participate are provided with a simple, remotely managed computer and internet connection. The set-up of the LISS panel in 2007 was funded by the Dutch Research Council and is managed by Centerdata, a non-profit institute housed at the campus of Tilburg University (The Netherlands). The number of invited (adult) panel members for the VICTIM-study varies between 6119 and 7096 across the years, and the response rate varies between 82.4 % and 87.9 %.

The data of the VICTIMS-study can efficiently be linked with archived data of all other (past or future) studies conducted with the LISS panel, such as the annual *Core studies* on Health and Personality, and the 300+ *Assembled studies* conducted with the LISS panel. This offers unique opportunities for researchers to address numerous research questions related to potential traumatic and stressful life-events.

Specifications Table

**Table:** 

Subject	Psychology
Specific subject area	Potentially traumatic events: PTSD, anxiety and depression symptoms; Social support; Physical, mental, family, work, financial, and religious problems; services use.
Data format	.SAV files (complete raw dataset of each survey^1^). .DTA files (complete raw dataset of each survey^1^). .CSV files (complete raw datasets of each survey, .csv since 2020 survey^1^) .PDF files (complete codebook, including routing of questions and metadata of each survey, available in English and Dutch). ^1^ In the raw data sets, answers to the open questions that contained information that may identify respondents were anonymized.
Type of data	Table
Data collection	Data were annually collected in the same months (March-April) from 2018 to 2023 using the online LISS panel, administered by Centerdata, the Netherlands. The panel is based on a large representative sample drawn from the Dutch population register by Statistics Netherlands (N∼7500). Participants gave explicit consent for the use of the data for scientific research. The surveys consist of standardized questionnaires on 21 potential traumatic and stressful events, mental health (PCL-5 8-item, MHI-5), lack of social support (SSL-D), social acknowledgement (SAQ), coping self-efficacy (CSE-7), problems and services use (PHIL).
Data source location	Centerdata, Tilburg, the Netherlands.
Data accessibility	Repository name: LISS panel data archive (In English): https://www.dataarchive.lissdata.nl/ Direct URL to data: 2018 survey: https://doi.org/10.17026/dans-zy7-9gfb 2019 survey: https://doi.org/10.17026/dans-zws-ffz7 2020 survey: https://doi.org/10.17026/dans-27v-paa3 2021 survey: https://doi.org/10.17026/dans-z73-b5j6 2022 survey: https://doi.org/10.57990/2n4q-pc88 2023 survey: https://doi.org/10.57990/p6bc-4e92 Background variables https://doi.org/10.57990/qn3k-as78
	Instructions for accessing these data: Centerdata is ISO 27001, NEN 7510, and ISO 9001 certified. The LISS Data Archive is CoreTrustSeal certified and free to browse. Before researchers can download datasets, they need to sign a statement on https://statements.centerdata.nl where the conditions of use are described. Importantly, access to data is for individual use only: every researcher who wants to use the data for research, individually or as researcher in a team of researchers, needs to register. It is prohibited for data users to make copies of the data available to others and/or archive the data in repositories.

## 1 Value of the Data

• The large multi-wave VICTIMS-study started in 2018 and is conducted with the Dutch *Longitudinal Internet studies for the Social Sciences* (LISS) panel. This panel is based on a large traditional probability sample of the Dutch population (16+) drawn by Statistics Netherlands from the population register (N∼7500). Importantly, panel members cannot register themselves in contrast to opt-in panels where members enroll themselves in the panel.
• The data of the six annual surveys are based on standardized validated questionnaires and questions that are derived from previous research on potential traumatic events. The annual surveys focus, among others, on potential traumatic events (PTEs) in the past 12 months and not on life-time exposure to PTEs. This was done to reduce the potential risk of biased recollections of PTEs as much as possible. In March 2024 a 7th similar survey will be conducted.
• The data of the six annual surveys of the VICTIMS-study can easily be merged with the archived data of the annual Core studies conducted in the LISS panel. A broad range of topics is included in these studies: Health, Religion and Ethnicity, Social Integration and Leisure, Family and Household, Work and Schooling, Personality, Politics and Values, and Economic Situation with subtopics such as Assets, Income, and Housing. In addition, all 300+ assembled cross-sectional and longitudinal studies conducted with the LISS panel since 2007 can be merged (as well as future Core and Assembled studies).
• The possibility to merge all data offers unique opportunities to conduct a wide range of prospective, longitudinal and cross-sectional studies on the determinants and effects of potential traumatic events, and their interplay. The available data create numerous opportunities to address gaps in scientific knowledge that are of relevance for improving support and interventions to minimize the negative effects of potentially traumatic events.
• Anxiety and depression symptomatology, lack of emotional and esteem support, problems and use of professional help, were assessed among all respondents (not only among respondents affected by PTEs) that participated in the annual surveys of the VICTIMS-study. The data on these variables may be of relevance for prospective, longitudinal and cross-sectional studies not related to potential traumatic events and their effects.
• The LISS data archive is user friendly and free to browse. Metadata is available in English and Dutch, and the data are labelled in English. Anyone can view the metadate online, can download the archived documentation (codebooks and questionnaires). After registration (accepting rules and regulations) all archived data files of interest can be downloaded for free. Data can be used for scientific, policy or socially relevant (i.e. non-commercial) research. All data can be merged using the variable nomem_encr that contains the encrypted individual panel member number. Because of the setup and routing of the online questions, missing values are minimal.

## 2 Data Description

### 2.1 Objective

The data are collected for the Dutch population-based multi-wave VICTIMS-study. In brief, the main aims are (1) to gather data to gain better insight in the patterns of exposure to potentially traumatic events, perceived social support and social acknowledgement, problems and services use, coping self-efficacy and mental health, and their interplay, (2) gather data that enables prospective longitudinal analyses among by recent potentially traumatic events (PTEs) affected adults compared to non-affected, using non-retrospective collected data on pre-existing factors [2,3], and (3) thereby contribute to the development of interventions and policies to minimize the negative effects of potentially traumatic events.

The VICTIMS-study was conducted using the population-based *Longitudinal Internet studies for the Social Sciences* (LISS) panel, that started in 2007 [1]. A special reason to use this panel was that the data on an individual level can be merged with the data of all past, current and future annual core studies and assembled studies (300+) that focus on other topics such as personality, work, religion, leisure, politics, and values, creating numerous opportunities for scientific research.

### 2.2 Overview Content and Order of Topics in Surveys

In Table 1 a brief overview is presented of the topics/questions that are similar across all six annual surveys of the VICTIMS-study. The topics/questions in *italics* were only administered to respondents who reported to be exposed to one or more potential traumatic events (PTEs) in the past 12 months.

**Table 1:** Brief overview content annual surveys VICTIMS-study.

Topic	Instrument
Lack of social support	
- Lack of emotional support	SSL-D
- Lack of esteem support	SSL-D
Problems and use professional help for these problems	
- Eight problems varying from physical to legal problems	PHIL
- Use of professional support for these problems	PHIL
21 Potential traumatic (PTE) and stressful life-events (SLE) in past 12 months:	
- Serious threat(s), without the use of physical violence (not *via* the internet)	Prev.
- Serious threat(s) online, without the use of physical violence (*via* the internet)	Prev.
- Sexual violence/sexual abuse (not *via* the internet)	Prev.
- Online sexual violence/sexual abuse (*via* the internet)	Prev.
- Robbery	Prev.
- Traffic accident	Prev.
- Airplane accident	Prev.
- Company accident	Prev.
- Fire	Prev.
- Burglary	Prev.
- Physical violence, not by own partner	Prev.
- Physical violence by own partner	Prev.
- Theft/fraud (not *via* the internet)	Prev.
- Online theft/fraud (*via* the internet)	Prev.
- Medical accident/error	Prev.
- Contraction of a serious infection (e.g. HIV, AIDS)	Prev.
- Development of a serious physical ailment (e.g. cancer, heart attack)	Prev.
- Death loved one (e.g. partner, family member, friend), expected^1^	Prev.
- Death loved one (e.g. partner, family member, friend), unexpected^1^	Prev.
- Death colleague, expected^1^	Prev.
- Death colleague, unexpected^1^	Prev.
- Other drastic or traumatic event (open question)	
- *In case of two or more events, which event most stressful*	Prev.
- *Time of (most stressful) event*	Prev.
- *Stress during (most stressful events)*	Prev.
- *Regarding (most stressful) event talk to or have any contact with:*	
*Nineteen categories of people varying from family to police*	Dev.
- *In case person did not speak to or have contact with anyone:*	–
*Did anyone knew what happened*	Dev.
- *Coping self-efficacy related to the (most stressful) event*	CSE-7
- *Posttraumatic stress symptoms related to (most stressful) event*	PCL5 8 item
- *Social acknowledgement related to the (most stressful) event*	SAQ
- *Social recognition*	–
- *General disapproval*	–
- *Family disapproval*	–
Anxiety and depression symptoms	MHI-5
Experience with PTE/SLE in past 12 months of people with whom respondent interacts	
- Five (2018)/ten (2019–023) different categories of events	Dev.
Evaluation survey	
- Six valuative questions varying from clarity to burdensome of survey	Std.

1 In the 2021 survey a distinction was made between COVID-19 pandemic related deaths and non-pandemic related deaths. SSL-D = Social Support List—Discrepancy. PHIL = Problems and Help Inventarisation-List. Prev. = based on previous research on PTE/SLE events (see below). Dev. = developed for the present this study. CSE-7 = coping self-efficacy 7-item list. PCL5= PTSD Checklist 5 8-item version. SAQ = Social Acknowledgment Questionnaire. MHI-5 = Mental Health Inventory, 5-item. Std. = each survey conducted with the LISS panel ends with 5 identical evaluative questions (difficult, sufficiently clear, get thinking about things, interesting subject, enjoy) and for the present study one items was added (burdensome).

In the next paragraphs the administered standardized questionnaires and questions are described. To facilitate identification of these variables in the data sets, we added the last three digits of the variable names (var. xxx) that are similar across all six surveys (the first five letters and digits of the variable names refer to the specific survey and are similar across all items of the specific survey). The order of administered standardized questionnaires and questions below is similar to the order in the surveys.

### 2.3 Lack of Emotional and Esteem Support

The Social Support List—Discrepancy [4,5] was administered to examine perceived lack of emotional support (8 items) and lack of esteem support (6 items). Examples of items are “*comfort you*” and “*emphasise your strong points*” (emotional support: var. 007, 008, 010, 011, 015, 016, 018, 020; esteem support: 006, 012, 013, 014, 017, 019). We added one item “lend you a friendly ear” from another scale of the SSL-D (var. 009). The respondents are instructed to consider all the people with whom they interact, including all family members, friends, acquaintances, neighbors, colleagues, and so on. Respondents are asked to indicate for each of the 15 items to what extent these people show behavior that they wish would be otherwise on a 4-point scales (1 = I miss it, I would like it to happen more often; 2 = I don't really miss it, but it would be nice if it happened a bit more often; 3 = Just right, I would not want it to happen more or less often; 4 = It happens too often, it would be nice if it happened less often). Low total scores indicate a greater lack of support.

### 2.4 Problems and Use of Professional Support

Respondents were administered the brief Problems and Help Inventarisation-List (PHIL) to examine the presence of physical problems, psychological problems, problems relating to religion, problems at work, problems in the family/with own partner, financial problems, problems with administration, and legal problems (var. 021 to 028) [6]. Respondents could indicate whether they experienced these problems or not (1 = yes, 2 = no). If respondents reported that they had (a) specific problem(s), they were asked about their need for professional support for the specific problem(s) (var. 029-036). Examples of such professionals are the (family) doctor, lawyer, psychologist, financial expert, physiotherapist, victim support case-manager, psychiatrist, chaplain (priest, pastor, imam, rabbi), (company) social worker. Using a 5-point scale respondents could indicate the answer that best described them (1 = I do not need professional support for this; 2 = I am receiving professional support for this; 3 = I could use professional support for this, but am not seeking it; 4 = I cannot find / get any suitable professional support for this; 5 = I cannot afford to pay suitable professional support for this).

### 2.5 Potential Traumatic Events in Past 12 Months

In each survey potential traumatic events (PTEs) were examined by a list of 21 events (1 = yes, 2 = no), based on previous research on PTEs (var. 037-057) [7], [8], [9], [10]. This list included events such as severe threat without physical violence, events with physical violence, accidents, but also the (un)expected death of a significant other (partner, family, friend) based on Criterion A1 events in DSM-IV and/or events in the ICD-11 (see Table 1). The VICTIMS-study focused on adults and events in the past twelve months. PTEs such as adverse child experiences are therefore excluded. Participants could describe PTEs in the past twelve months that were not listed (var. 058-059).

If respondents experienced one PTE in the past 12 months, respondents were asked to keep this event in mind when answering the event-related questions (such as on stress during the PTE and PTSD symptoms). This event was displayed on the screen when event-related questions (about PTSD, coping self-efficacy, contacts with other, social acknowledgement) were administered (var. 002). In case respondents reported two or more events, respondents were asked to rate the most adverse or stressful one. When respondents had been confronted with a serious disease, (expected or unexpected) death of a significant other or colleague (var. 052-057) *and* a violence-, threat-, theft, or accident-related event respondents (var. 037-051) were automatically asked to keep the violence-, threat-, theft, or accident-related event in mind when answering the event-related questions such as stress during the event and PTSD symptoms (var. 060-061).

In the datasets, the data of var. 002 (the type of the (most drastic) event), is in Dutch. In appendix 1 the 21 events are translated in English and the SPSS syntax offered to recode the 21 events for research purposes (with 2023 as an example).

### 2.6 Timing and Experienced Stress During Event

Based on previous research [10], by PTE affected respondents were asked when the (most drastic or stressful) PTE in the past 12 months took place (1 = 1 week ago; 2 = 2 weeks ago, 3 = 3 weeks ago; 4 = 4 weeks ago; 5 = 1–2 month(s) ago; 6 = 3–4 months ago; 7 = 5–6 months ago; 8 = 7–12 months ago; var. 062). In addition, respondents were asked to indicate how much tension or stress the event caused (at the time that it happened, so not at the present moment) on a 5-point Likert scale (1 = Not at all or barely; 2 = A little bit; 3 = Moderately; 4 = Quite a bit; 5 = Extremely)(var. 063).

### 2.7 Contact with Others About Event

In addition, by PTE affected respondents were asked to indicate if they talked or had any contact with others regarding (the most stressful) event (1 = yes) or not (0 = no): family/friends/neighbors /acquaintances; family doctor/medical specialist; police: your (children's) school; chaplain, e.g., pastor, priest, rabbi, imam; employer/manager; colleagues/class or fellow students; therapist, psychiatrist, psychologist; public prosecutor; lawyer; judge; victim support; fellow sufferer/people who had the same experience; organization to counter domestic violence; social work; public health service/city government; alternative therapy sector; other organization; other people than listed above (var. 064 to 082).

They could also indicate that they did not speak to or have had no contact with anyone about the event (0 = no, 1 = yes)(var. 083). If that was the case, the following question was presented “*You have just indicated that you did not speak to or have contact with anyone about this event. We would like to know whether there were other people who nevertheless know that you experienced this event (for example because they saw or heard what happened, in one or other way). Does anyone know that you experienced this stressful event*?”. Answer categories were: 1= Yes, one or more persons; 2 = Yes, I suspect one or more persons; 3 = No, nobody at all (except the perpetrator, if applicable); and 4 = I don't know (var. 084).

### 2.8 Event-Related Coping Self-Efficacy

The 7-item Coping Self-Efficacy list (CSE-7) [11] was administered to assess current coping self-efficacy related to the (must stressful) event (var. 085 to 091). By PTE affected respondents were asked to estimate their capacity to handle tasks such as “*Dealing with the impact that the traumatic experience has had on my life*” and “*Seeking help from other people because of what happened*” described on a 7-point Likert scale (1 = I am entirely incapable, 4 = I am reasonably capable; 7 = I am completely capable). Higher total scores indicate higher coping self-efficacy levels.

### 2.9 Posttraumatic Stress Symptoms

PTSD-symptomatology following the (most stressful or selected) PTE among affected respondents was assessed using the 8-items version of the PCL-5 [12], [13], [14] that assessed symptoms across the four symptom clusters of PTSD according to DSM-5 (APA, 2013; var. 092 to 099). Example of PCL5 items are “ *Feeling very upset when something reminded you of the stressful experience*” and “*Loss of interest in activities that you used to enjoy*”. Items have 5-point Likert scales and focus on symptoms in the past month (1= Not at all; 2 = A little bit; 3 = Moderately; 4 = Quite a bit; and 5 = Extremely). For the sum scores, all values have to be recoded as follows: 1 = 0, 2 = 1, 3 = 2, 4 = 3, 5 = 4.

### 2.10 Social Acknowledgement

Event-related social acknowledgement was examined by the Social Acknowledgement Questionnaire (SAQ) [15,16]. The SAQ consists of the scales Event-related recognition as victim (5 items, var. 100 to 104), general disapproval (6 items, var. 105 to 109, 115), and family disapproval (5 items, var. 110-114). The SAQ has positively and negatively formulated items (statements) with 5-point answer categories (1 = totally disagree, 2 = agree, 3 = neutral; 4 = agree; 5 = totally agree, 6 = not applicable). Examples of items are “*Most people cannot understand what I went through*”, “*The reactions of my acquaintances were helpful*” and “*My family showed a lot of understanding for my state after the incident*”.

### 2.11 Anxiety and Depression Symptoms

The 5-item Mental Health Inventory [17,18] was administered to examine anxiety and depression symptomatology (var. 116 to 120). The MHI-5 asks respondents to rate their mental health during the past month on 6-point Likert scales (1 = never; 2 = rarely; 3 = sometimes; 4 = often; 5 = mostly; 6 = permanently). Examples or items are “*I was very nervous*” and “*I felt so down that nothing could cheer me up*”. To obtain a total score, the negatively formulated items have to be inversely recoded first. Next, all values have to be recoded as follows: 1 = 0, 2 = 1, 3 = 2, 4 = 3, 5 = 4, 6 =5. The total score has to be multiplied by 4 to obtain a score range of 0-100. Lower scores indicate higher anxiety and depression symptomatology levels.

### 2.12 Potential Traumatic Events in Past 12 Months Among Significant Others

Respondents were asked if any of the people with whom they interact (family members, friends, acquaintances, neighbors, colleagues, and so on) experienced one or more potentially traumatic events in the past 12 months (answer categories 1 = yes; 2 =no). In the 2018 survey five categories were distinguished: (1.) Violence/burglary/robbery/sexual violence or abuse, (2.) accident/fire/disaster, serious sickness/serious infection/medical error, (3.) Fraud/internet fraud, (4.) Death of a loved one (e.g. partner, family, friend, colleague), (5.) and other (0 = 0, 1 = yes; var. 121, 122 to 127c). In the 2019 to 2023 surveys 10 categories were distinguished (1.) serious threat, (2.) traffic accident; (3.) other accident, calamity or disaster, (4.) physical violence (not by the (former) partner of the person concerned, (5.) physical violence, by the (former) partner of the person concerned, (6.) sexual violence/rape/sexual abuse, (7.) sexual harassment/sexual abuse via the internet, (8.) theft/fraud (*via* the internet or not), (9.) burglary in the home, and (10.) medical accident/error (1 = yes, 2 = no)(var. 158 to 167).

### 2.13 Evaluation Survey

Each survey, short or long, conducted with the LISS panel ends with 5 evaluation questions about the specific survey . Respondents are asked to rate the difficulty, clarity, reason to think about things, appealing of subjects, and how much they enjoyed the questions on a 5-point Likert scale (1 = certainly not to 5 = most certainly; var. 145-149). We added one item to the standard list “*Did you find the questionnaire burdensome?*” with the same Likert-scale (var. 150).

### 2.14 Data of Surveys Collected for the VICTIMS-Study

The data of the surveys are archived in the LISS data archive that can be found at https://www.dataarchive.lissdata.nl/study_units/view/1. The opening page provides an overview of all annual *Core studies* and *Assembled studies* (300+) conducted since 2007, and the *Background variables* of the respondents of which the data are already archived and available

The complete raw datafiles and documentation of each survey of the VICTIMS-study can be found at https://www.dataarchive.lissdata.nl/study_units/view/945 (Assembled study 208). Under the heading *List of measures* interested researchers can select a survey (wave) of the VICTIMS-study to access the metadata of the specific survey (see also DOIs’ mentioned in **Data accessibility)**. After selecting the survey of interest, a webpage with the following headings (information and data) is presented:

- **General Information**, with subheadings Title, Project Number, Abstract, Longitudinal Type, Begin date, End date, Researcher, Publisher, Copyright, Funding Organization, and DOI.
- **Datasets and documentation**, with subheadings *View documentation* (codebook in English, codebook in Dutch) and *Data Files* (English SPSS file, English STATA file, English CSV file (CSV file since 2020)).
- **Variables** (Variable names, Variable Labels and Variable Types).
- **Questions** (In English and Dutch: Question name, Version, Question text, Question Type).
- **Response information**, with the subheadings *Response Overview* (Selected number of household members, Non-response, Response, Complete, Incomplete) and *Collection events* (Period, Sample, Collection mode, Fieldwork Note).

Registered users can download the dataset of the survey of interest (and/or download other datasets of this study, and datasets of all *Core* and *Assembled studies*).

### 2.15 Data Demographics Respondents

The demographic characteristics of the respondents at the time they participated in a specific survey of the VICTIMS-study (such as age, gender, primary occupation, and marital status) are *not* included in the datasets of the VICTIMS-study (nor in datasets of other studies). They are archived in the Background Variables part of the LISS data archive that can be found at https://www.dataarchive.lissdata.nl/study_units/view/322 (Background variables; see also DOI mentioned in **Data accessibility**). After selecting *Background Variables* a webpage with the following headings is presented:

- **General information** (Title, project number, Abstract, Longitudinal Type, Researcher, Publisher, Copyright, Funding Organization).
- **Datasets and documentation**, with subheadings *View documentation* (codebook in English, codebook in Dutch; imputation procedure missing income information) and *Data Files* (separate files for each month since the start of the LISS panel in 2007).

The Background Variables can easily be merged with the data of a survey of the VICTIMS-study (or other study) using the *key variable* nomem_encr that contains all encrypted individual codes of the respondents. For instance, to merge data of respondents who filled in the questions of the VICTIMS-study in March 2021 with the data of Background Variables of the same month, registered users select and merge the Data file *English March2021* (avars_202103_EN_1.0p.zip).

With the key variable nomem_encr the data can also be merged with the data of other surveys in the LISS data archive.

Table 2 provides an overview of the response and sample characteristics (of the respondents of 18 years and older) weighted for the Dutch adult population. The applied weights are based on the open access data of Statistics Netherlands (CBS, https://opendata.cbs.nl/statline/#/CBS/en/) on gender, age (18 years or older) and marital status of the year in which the surveys were conducted. In Appendix 2, the extracted numbers of males and females (total), married males and females of 18 years and older in the year 2018 up to 2023 are presented.

**Table 2:** Response, sample characteristics and applied weighting statistics of the six annual surveys of the VICTIMS-study.

Year survey^1^	2018^3^	2019^3^	2020	2021	2022	2023
**Response**						
N^invited^	7292	6298	6568	6452	6737	6409
N Response (%)	6012 (82.4)	5250 (83.4)	5773 (87.9)	5608 (86.9)	5644 (83.8)	5281 (82.4)
**Sample characteristics**						
Gender	*n* (%)	*n* (%)	*n* (%)	*n* (%)	*n* (%)	*n* (%)
- males	2895 (49.2)	2526 (49.3)	2847 (49.3)	2767 (49.3)	2785 (49.3)	2603 (49.3)
- females	2984 (50.8)	2600 (50.7)	2926 (50.7)	2840 (50.7)	2856 (50.6)	2674 (50.6)
- other					3 (0.05)	4 (0.08)
Age						
- 18–24	636 (10.8)	557 (10.9)	629 (10.9)	610 (10.9)	622 (11.0)	586 (11.1)
- 25–34	920 (15.7)	809 (15.8)	920 (15.9)	894 (15.9)	902 (16.0)	852 (16.1)
- 35–44	879 (15.0)	757 (14.8)	848 (14.7)	826 (14.7)	835 (14.8)	790 (15.0)
- 45–54	1086 (18.5)	925 (18.0)	1013 (17.5)	957 (17.1)	936 (16.6)	851 (16.1)
- 55–64	976 (16.6)	857 (16.7)	972 (16.8)	951 (17.0)	957 (17.0)	891 (16.9)
- 65–74	798 (13.6)	702 (13.7)	795 (13.8)	782 (13.9)	773 (13.7)	713 (13.5)
- 75–84	425 (7.2)	379 (7.4)	439 (7.6)	433 (7.7)	462 (8.2)	452 (8.6)
- 85 and older	158 (2.7)	139 (2.7)	158 (2.7)	154 (2.7)	156 (2.8)	146 (2.8)
Civil status						
- married	2860 (48.6)	2469 (48.2)	2754 (47.7)	2644 (47.2)	2632 (46.6)	2442 (46.2)
- divorced	541 (9.2)	469 (9.1)	546 (9.5)	563 (10.0)	580 (10.3)	517 (9.8)
- widow	348 (5.9)	315 (6.1)	337 (5.8)	314 (5.6)	312 (5.5)	299 (5.7)
- never married	2130 (36.2)	1873 (36.5)	2135 (37.0)	2086 (37.2)	2121 (37.6)	2023 (38.3)
Education level						
- primary school	393 (6.7)	346 (6.7)	362 (6.3)	371 (6.6)	349 (6.2)	339 (6.4)
- interm. sec. edu.	1119 (19.0)	953 (18.6)	1020 (17.7)	947 (16.9)	897 (15.9)	818 (15.5)
- higher sec. edu.	734 (12.5)	640 (12.5)	679 (11.8)	660 (11.8)	651 (11.5)	604 (11.4)
- interm. voc. edu.	1401 (23.8)	1196 (23.3)	1418 (24.6)	1366 (24.4)	1401 (24.8)	1296 (24.5)
- higher voc. edu.	1454 (24.7)	1284 (25.0)	1486 (25.7)	1471 (26.2)	1500 (26.6)	1429 (27.1)
- university	777 (13.2)	708 (13.8)	808 (14.0)	791 (14.1)	845 (15.0)	795 (15.1)
Primary occupation						
- employed	3116 (53.0)	2754 (53.7)	3157 (54.7)	3049 (54.4)	3140 (55.6)	2979 (56.4)
- job seeker	169 (2.9)	121 (2.4)	126 (2.2)	120 (2.1)	76 (1.3)	70 (1.3)
- student	578 (9.8)	489 (9.5)	496 (8.6)	502 (9.0)	490 (8.7)	466 (8.8)
- household	442 (7.5)	396 (7.7)	431 (7.5)	413 (7.4)	405 (7.2)	360 (6.8)
- pension	1147 (19.5)	984 (19.2)	1107 (19.2)	1110 (19.8)	1140 (20.2)	1044 (19.8)
- (partial) work disabled	246 (4.2)	220 (4.3)	252 (4.4)	235 (4.2)	234 (4.1)	204 (3.9)
- volunteer work	121 (2.1)	103 (2.0)	116 (2.0)	102 (1.8)	96 (1.7)	85 (1.6)
- other	62 (1.1)	58 (1.1)	88 (1.5)	76 (1.4)	64 (1.1)	73 (1.4)
**Weight descriptives statistics**						
- Mean (SD)	1.09 (0.45)	1.13 (0.48)	1.08 (0.41)	1.12 (0.56)	1.09 (0.42)	1.14 (0.60)
- lowest	0.51	0.47	0.46	0.46	0.49	0.45
- largest	3.50	2.89	2.78	4.33	2.36	3.99
- median	1.02	1.06	1.01	1.01	1.04	1.04

1 Surveys were conducted in March, and repeated to nonrespondents in April. Reminders were sent twice each month. ^2^Data are weighted for the adult Dutch population of the same year (see Appendix 1).

3 This survey was also administered to 16 and 17 year old panel members and but were excluded from the sample characterics in this table (N^2018^ = 196, N^2019^ = 179).

Based on the extracted CBS data of each year we composed 56 exclusive demographic profiles of each year as follows. We first divided age in 14 age categories (18–24, 25–29, 30–34, 35–39, 40–44, 45–49, 50–54, 55–59, 60–64, 65–69, 70–74, 75–79, 80–84, 85 years or older). In each age category we next distinguished four exclusive subgroups based on gender and marital status (males married, males unmarried, females married, females unmarried) totaling 14*2*2=56 demographic profiles in each year. The distribution of the 56 profiles of a specific year was used to compute the weights for the data of the survey of that year. In the lower part of Table 2 the weight descriptive statistics are presented. To prevent a lengthy table, the age categories were reduced to 7 categories in Table 2.

**Education level.** Primary school = primary school including unknown. Interm. sec. edu = intermediate secondary education, US: junior high school, NL= VMBO. Higher sec. edu. = higher secondary education/preparatory university education, US: senior high school, NL=HAVO, VWO. Interm. voc. edu. = intermediate vocational education, US: junior college, NL: MBO. Higher voc. edu. = higher vocational education, US: college, NL: HBO.

**Primary occupation.** Employed = paid employment, works or assists in family business, autonomous professional, freelancer, or self-employed. Volunteer work = performs unpaid work while retaining unemployment benefit, performs voluntary work. Job seeker = Job seeker following job loss, first-time job seeker. Other = exempted from job seeking following job loss, soes something else.

For an overview of published studies based on data of the VICTIMS-study (until October 2023) see https://www.dataarchive.lissdata.nl/study_units/view/941.

## 3 Experimental Design, Materials and Methods

As described above, the surveys of the VICTIMS-study were conducted with the Dutch *Longitudinal Internet studies for the Social Sciences* (LISS) panel. The set-up of the LISS panel in 2007 was funded by the Dutch Research Council (NWO) and the panel is managed by Centerdata. Panel members receive an incentive of 15 euros per hour and members who do not have a computer and/or internet access are provided with the necessary equipment at home for free.

### 3.1 Recruitment LISS Panel

For the main recruitment wave of the LISS panel at the start in 2007, a random sample of 10,150 addresses was drawn by Statistics Netherlands (CBS) from the population register. Recruitment of the sampled households was done from May until December 2007 by the fieldwork institute TNS NIPO. The households were contacted in a traditional manner: first an announcement letter was sent along with a brochure explaining the nature of the panel study. The envelope also contained a 10 euro banknote. Next, respondents were approached by an interviewer in a mixed mode design. Households with a known telephone number were contacted by phone (Computer Assisted Telephone Interviewing, CATI). The other households were visited by an interviewer and thus contacted face-to-face (Computer Assisted Personal Interviewing, CAPI). Once the households were contacted, the interviewer asked the respondents to participate in a 10 min interview, after which they were invited to participate in the panel. Respondents that agreed to participate in the panel were required to confirm their participation by registering on the panel website or by returning a signed response slip. In total, 75 % of the contacted persons were willing to participate of whom 84 % actually participated.

The recruitment of a first refreshment sample was carried out between June and December 2009. In cooperation with Statistics Netherlands, a stratified sample was drawn intended to improve the representativeness of the panel by oversampling the difficult-to-reach groups which had a below-average response in the main recruitment. The refreshment sample in 2009 was stratified on three variables: (1) household type, (2) age, (3) ethnicity. Between October 2011 and May 2012 a second refreshment sample for the LISS panel was recruited. This sample was randomly drawn from the population registers by Statistics Netherlands, in the same way as the original sample of 2007. Between November 2013 and June 2014 a third refreshment sample was recruited (stratified). Between November 2016 and June 2017 the fourth refreshment sample was recruited (again, stratified as in 2009 and 2013). The fifth refreshment sample was recruited between October 2019 and August 2020, also based on a stratified sample. During this fifth refreshment round, it became clear that households were harder to reach *via* CATI mode. As a result, a greater number of households had to be recruited through CAPI. Consequently, the fieldwork period had to be extended compared to previous refreshment rounds. The sixth refreshment round, which occurred between February 2022 and August 2022, was based on a random sample using a target group oriented approach. In this round, households were contacted sequentially in CAWI-CATI-CAPI-PAPI (Paper-and-Pencil Interviewing) modes (instead of CATI-CAPI, as used in all earlier refreshment rounds). By contacting households through CAWI as the first mode, the initial response rate saw a significant improvement.

### 3.2 Timing Surveys

The online surveys of the VICTIMS-study were administered in March of 2018, 2019, 2020, 2021, 2022 and 2023. Reminders of the surveys were sent to the non-responders one month later in April of each year. The next four surveys are scheduled for March 2024, 2025, 2026 and 2027.

In total and across the six surveys, 3051 panel members participated in all six surveys, 3774 in five or more surveys, 4758 in four or more, 5625 in three or more, 7162 in two or more, and 1894 in only one survey.

In October 2018, among the subsample of adult respondents affected by (1.) burglary, theft or fraud; (2) accidents (including traffic accidents, medical accidents/error, other accidents or disaster; or (3) serious threat or (sexual) violence in 12 months before the March 2018 survey, current posttraumatic stress symptoms and social acknowledgement were assessed (follow-up measurement 2018: N^invited^ = 720, response = 88.3 %). Because this was the only follow-up measurement among a selective sub-sample we refer to Van der Velden et al. [19] for further information about the data of this survey.

## Limitations

The LISS panel does not include respondents younger than 16 years old, and residents that do not speak and cannot read Dutch. We did not conduct clinical interviews to examine mental disorders such as PTSD, major depression, generalized anxiety disorder among the respondents. The answers to the open question which other events respondents were exposed to, were not translated into English.

## Ethics Statement

In accordance with the General Data Protection Regulation (GDPR), participants gave explicit digital consent for the use of the collected data for scientific and policy relevant research. The VICTIMS-study and questionnaire was approved by an Internal Review Board of Centerdata, consisting of independent, internal and external reviewers. These reviewers were not involved in the development of the VICTIMS-study. The authors assert that all procedures contributing to this work comply with the ethical standards of the relevant national and institutional committees on human experimentation and with the Helsinki Declaration of 1975, as revised in 2008. Since our research did not impose certain (experimental) behaviour, our research did not need the approval of a Dutch Medical Ethical Testing committee according to the Dutch Law (see https://english.ccmo.nl/). Importantly, previous research using this panel revealed that the possible burden of participating in research on trauma was related to the perceived burden of other nontrauma studies such as on political values, but not with PTSD symptoms or other trauma-related variables [10]. With respect to data security, Centerdata is ISO 27001 and NEN 7510 certified. The LISS data archive obtained the CoreTrustSeal certification.

## CRediT authorship contribution statement

**Peter G. van der Velden:** Conceptualization, Methodology, Investigation, Data curation, Formal analysis, Writing – original draft, Writing – review & editing, Funding acquisition, Project administration, Supervision. **Carlo Contino:** Conceptualization, Methodology, Writing – review & editing. **Marcel Das:** Conceptualization, Methodology, Investigation, Formal analysis, Writing – review & editing, Funding acquisition.

## Data Availability

Victims in Modern Society (assembled study 208 (Original data) (https://www.dataarchive.lissdata.nl/study_units/view/941)

## Appendix Supplementary materials

Image, application 1

Image, application 2
